# Effect of Electrode Surface Microstructuring on Electrochemical Biosensor Performance

**DOI:** 10.3390/ma18071390

**Published:** 2025-03-21

**Authors:** Amal Kabalan, Maliheh Azimi Roueini

**Affiliations:** Department of Electrical and Computer Engineering, Bucknell University, Lewisburg, PA 17837, USA; mar059@bucknell.edu

**Keywords:** electrochemical sensors, microstructuring, adsorption

## Abstract

Electrode surface microstructuring involves the engineering of the topographical features of an electrode to enhance its performance in electrochemical sensing applications. By creating controlled micro- or nano-scale patterns, the active surface area can significantly increase, which leads to improved electron transfer and enhanced sensitivity to target analytes in devices such as biosensors. Geometrical parameters such as diameter, height, pitch, and position of the patterns can be optimized to enhance sensor detection. This paper introduces an electrochemical biosensor designed to detect *Moraxella catarrhalis*, a respiratory pathogen affecting young children. This paper investigates the effects of the radius of micropillars on adsorption in the electrochemical biosensor using COMSOL Multiphysics (Version: 6.0). The model demonstrates that the rate of surface adsorption depends on the position of the micropillars on the electrode. The paper also presents the effects of analyte concentration on the detection current of the biosensor using Cottrell’s equation.

## 1. Introduction

Electrochemical biosensors are analytical devices that convert a biological event—such as the binding of an enzyme, antibody, or other biorecognition element to a target molecule—into an electrical signal [1]. This signal can be measured in terms of current, voltage, or impedance and provides valuable information about the presence or concentration of specific analytes [1]. These sensors are characterized by high sensitivity and rapid response, which makes them attractive to applications in a wide variety of fields [2,3]. In medical diagnostics, they enable rapid point-of-care testing for biomarkers of diseases such as diabetes, cancer, and infectious pathogens, thereby facilitating timely clinical decisions [4]. In food safety, these sensors detect contaminants like pathogens, allergens, and chemical residues, ensuring product quality and consumer health. Environmental monitoring is another key area where electrochemical biosensors are used to measure pollutants, heavy metals, and other hazardous substances in water and soil, helping maintain ecological balance [5]. Advancements in electrode fabrication, such as micro- and nano-structuring, further enhance these sensors’ performance by increasing the surface area, improving mass transport, and facilitating targeted surface functionalization [6].

In this study, we introduce an electrochemical biosensor that aims to detect M. Catarrhalis, which is a pathogen that causes ear infections, especially in young children [7,8,9]. Specifically, this paper investigates the effects of the radius of micropillars on the adsorption rate in the electrochemical biosensor using COMSOL Multiphysics (Version: 6.0) [10]. It also analyzes the adsorption rate with respect to the position of the micropillar on the electrode surface.

## 2. Materials and Methods

**Figure 1:**
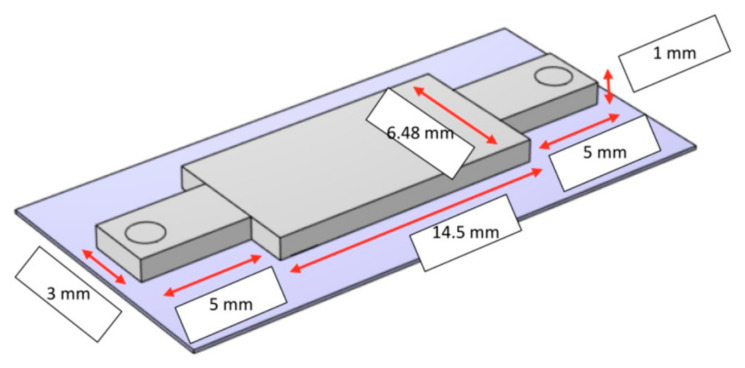
The sensing platform of the device [1].

**Figure 2:**
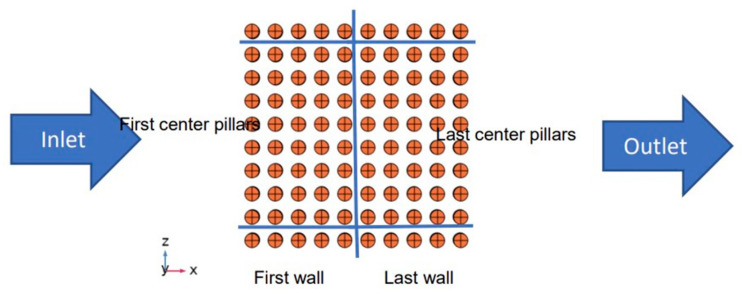
Patterned working electrode in the biosensor.

The sensing platform of the device is encapsulated in a microfluidic channel, as shown in Figure 1, and is composed of three electrodes (reference, working, and counter electrodes). The adsorption of the pathogen takes place at the working electrode, which is patterned by micropillars and shown in Figure 2. The surface of the working electrode is functionalized with antibodies to bond with the pathogen in a given fluid. The shape and the position of the micropillars impact the adsorption rate, which is analyzed in this study.

Analyte molecules (P—in this case, *Moraxella catarrhalis*) can adsorb and desorb from surface sites (S) on the micropillar’s surfaces according to Equation (1):

(1) $$\begin{array}{c} k_{\mathrm{ads}}\\ p+s\;↔\;\;ps \end{array}$$

The adsorbed analyte (*Moraxella catarrhalis*) of Equation (1) can transform into a quenched state (QS) that does not contribute to the sensor signal, as shown in Equation (2).

(2) $$\begin{array}{c} K_{1}\\ PS\;\;↔\;QS\;\;\\ K_{2} \end{array}$$

The rate of adsorption depends on the concentration of the analyte species in its stream. The equations describe the surface reactions that are coupled to free analyte flow. The movement of analyte molecules in a biosensor is due to the lateral flow in the device [11].

## 3. Results

**Figure 3:**
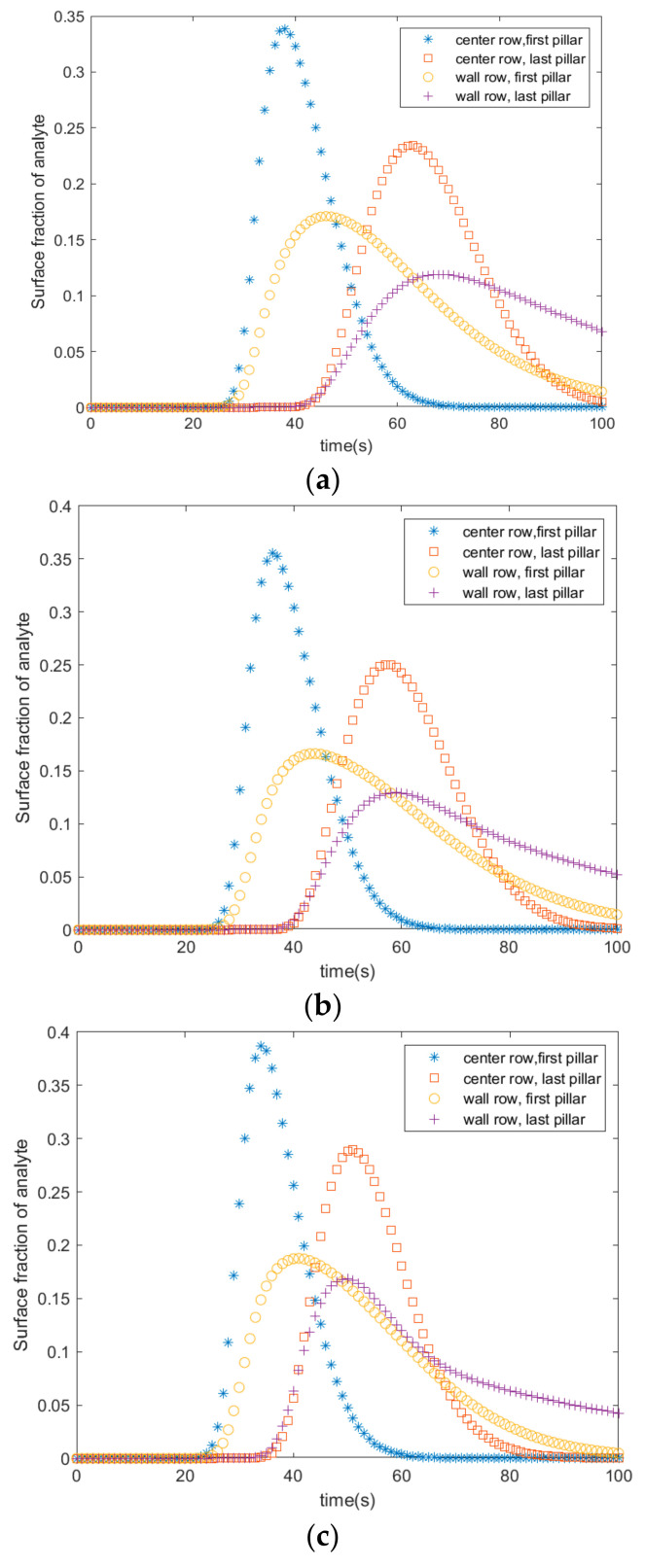
Average fractional surface coverage of adsorbed analyte in pillars with radii of (**a**) 0.3 mm, (**b**) 0.4 mm, and (**c**) 0.5 mm.

The rate of adsorption with respect to the position of the pillars in the microfluidic channel was studied using COMSOL Multiphysics. Figure 3 shows the rate of adsorption at four different locations of the pillars for three different radii (0.3 mm, 0.4 mm, and 0.5 mm). The following references were consulted when choosing the dimensions of the pillars [11,12]. Adsorption starts 25 s after the fluid enters the channel. It starts at the first pillar in the center and wall rows. It peaks at 33% after 35 s in the center row, and it peaks at 16% after 42 s in the wall row. Adsorption starts at 43 s in the last pillars of the wall and first rows. The adsorption peaks at 24% and 10% after 70 s in the last pillars of the wall and center rows, respectively.

**Figure 4:**
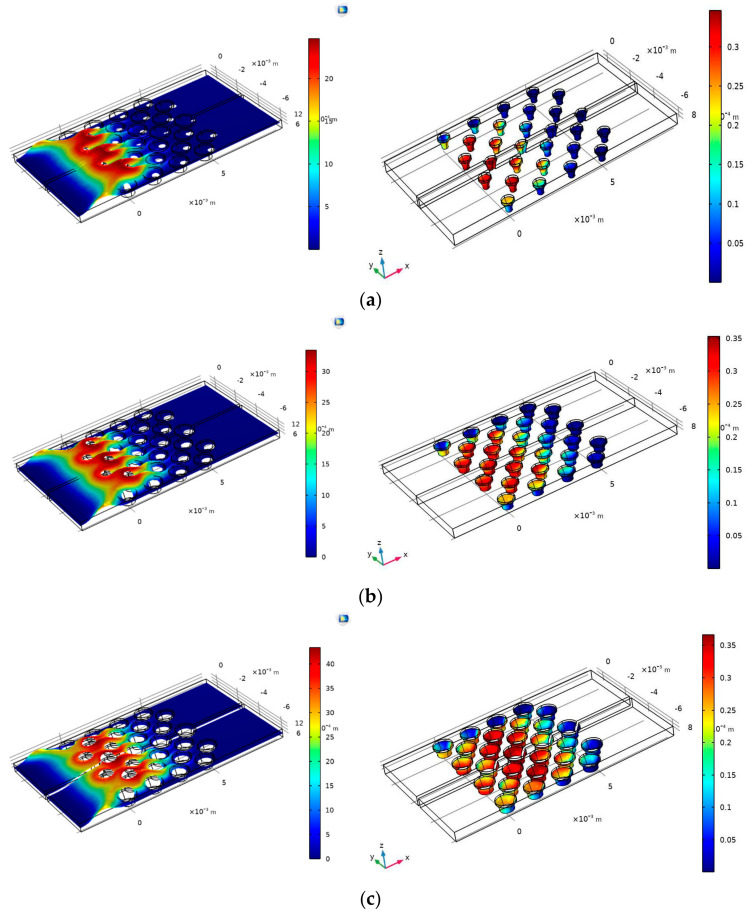
The average fractional surface coverage of adsorbed analytes shown on the gradient scale in pillars with a 0.3 mm radius: (**a**) 0.4 mm radius (**b**), and 0.5 mm radius (**c**). The left-hand images indicate the laminar flow in each channel.

**Figure 5:**
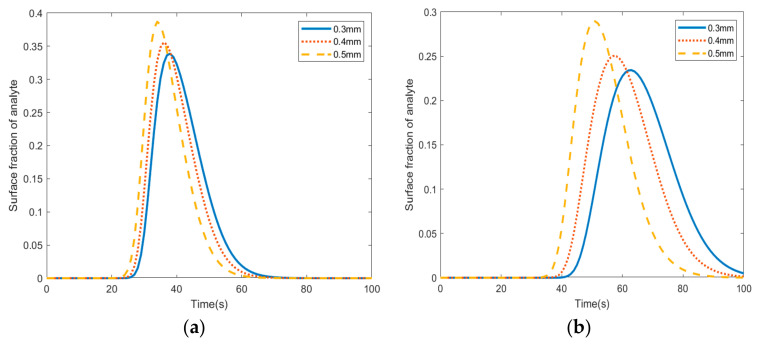
The average fractional surface coverage of the adsorbed analyte in pillars (0.3 mm, 0.4 mm, and 0.5 mm radii) located in the (**a**) center of the electrode and (**b**) pillars located at the edges of the electrode.

Figure 4a–c show the velocity (left) and adsorption fraction (right) in three micropillar radii (0.3 mm, 0.4 mm, and 0.5 mm) based on the location of the micropillar in the microfluidic channel. The data were obtained at t = 70 s (where t = 0 is the time the fluid enters the microfluidic channel). It was observed that as the radius of the pillars increased the adsorption fraction increased among the pillars in the channel. In Figure 4a, micropillars located at coordinates (0, −5), (0, −3), (1, −5), (1, −4), and (1, −2) have the highest absorption at t = 70 s. This pattern continues in Figure 4b, where the radius is set to 0.4 mm. Figure 4c shows that the highest adsorption occurs in the center pillars at coordinates (3, −6), (4, −6), and (4, −4). Figure 5 depicts the concentration distribution in the analyte (*Moraxella catarrhalis*) stream and the surface coverage of the adsorbed species in various radii. The comparison of Figure 5a,b show that pillars located at the center of the electrode (Figure 5a) have higher adsorption than center micropillars (Figure 5b).

**Figure 6:**
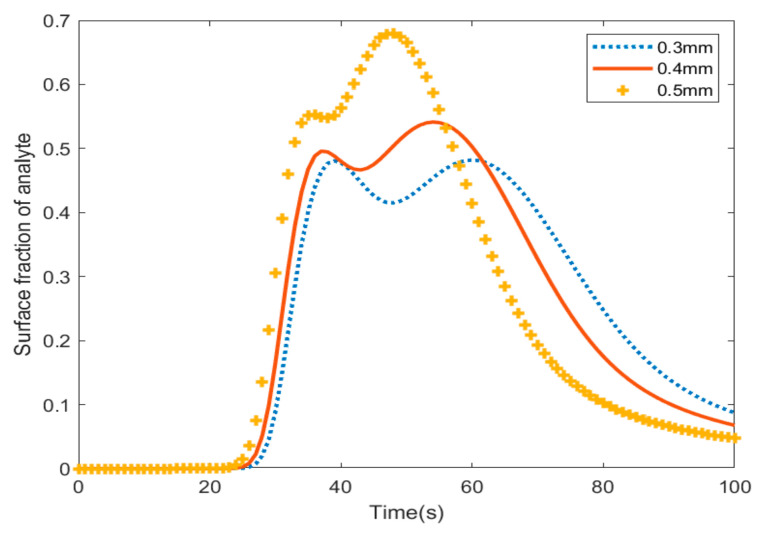
The total surface adsorption of the micropillars in the microfluidic channel.

Figure 6 shows the fraction of analyte absorption with varying radii of pillars in the channel. Based on the results in Figure 4, at t = 25 s, the sum of the adsorption of the analyte on pillars with a radius of 0.5 mm is higher than the other sizes; however, after 58 s, the biosensor that has pillars with the largest radius shows the lowest amount of adsorption. In other words, the largest pillars have the most adsorption and they desorb analytes quicker than other pillars.

**Figure 7:**
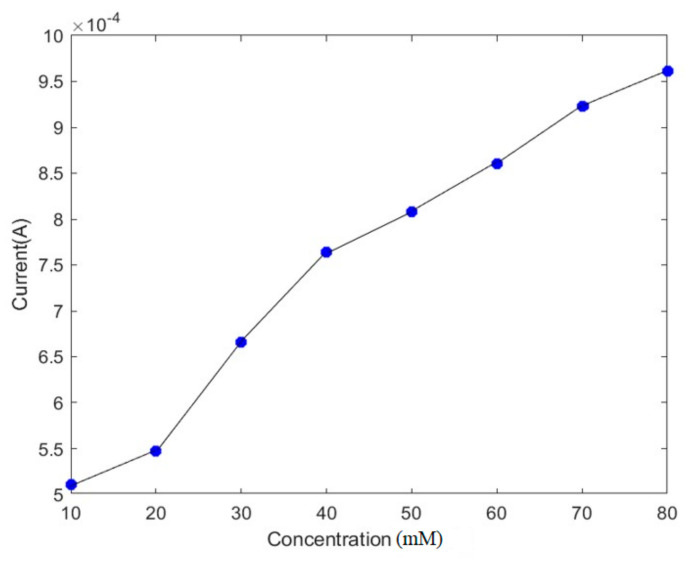
The effect of the pathogen concentration on the current.

We also investigated the changes in the current by varying the concentrations of the analyte. The current in the electrochemical biosensors can be calculated using the Cottrell equation [13]:

(3) $$i=nFcA\sqrt{D/\pi t}$$

where i is the current density, n is the number of electrons transferred per molecule of the analyte, c is the bulk concentration of the analyte, D is its diffusion coefficient, A is the area, and F is Faraday’s constant [14]. We varied the bulk concentration between 10 and 80 mM and investigated its effects on the current. Figure 7 shows that the absolute value of the response current increased proportionally with the increase in the analyte. The value of the current varied from 0.5 mA to 1 mA.

## 4. Conclusions

In this study, a Multiphysics computational model was developed to study the effects of the micropillar radius on the absorption of the pathogen M. Catarrhalis under laminar flow. It was observed that the rate of adsorption varies based on the location of the micropillar within the microfluidic channel. Micropillars in the center of the channel exhibit the highest absorption rates. The increased radius size also contributes to an increase in the surface area, which increases absorption. In addition, the effects of the concentration of the analyte on the current over time were analyzed based on the Cottrell equation. Understanding the effects of the concentration of the analyte on the electric current response plays a critical role in the design parameters of electrochemical biosensors. The next step is to further optimize the parameters of the microfluidic channel and consider the effect of turbulence flow on the device.

## Data Availability

The original contributions presented in this study are included in the article. Further inquiries can be directed to the corresponding authors.
